# A retrospective cohort study of clinical characteristics and healthcare contacts in Sweden prior to suicide in individuals with heart disease

**DOI:** 10.1186/s12875-026-03184-x

**Published:** 2026-02-09

**Authors:** Nina Palmqvist Öberg, Sara Lindström, Erik Bergqvist, Anna Ehnvall, Marjan Vaez, Tabita Sellin, Charlotta Sunnqvist, Margda Waern, Åsa Westrin

**Affiliations:** 1 Department of Clinical Sciences, Unit for Suicide Research, Lund, Psychiatry, Lund University, Lund, SE-221 84 Sweden; 2 Department of Psychiatry, Skåne University Hospital, Lund, SE-221 85 Sweden; 3 Psychiatric In-patient Clinic, Hallands Sjukhus Varberg, Varberg, Region Halland SE-432 81 Sweden; 4 Department of Psychiatry and Neurochemistry, Institute of Neuroscience and Physiology, University of Gothenburg, Gothenburg, SE-413 45 Sweden; 5 Psychiatric Out-patient Clinic, Varberg, SE-432 43 Region Halland Sweden; 6 Department of Clinical Neuroscience, Division of Insurance Medicine, Karolinska Institutet, Stockholm, 171 77 Sweden; 7 Faculty of Medicine and Health, University Health Care Research Center, Örebro University, Örebro, SE-701 82 Sweden; 8 The Region Skåne Committee on Psychiatriy, Habilitation and Technical Aids, Lund, Sweden; 9 Psychosis Clinic, Sahlgrenska University Hospital, Gothenburg, Region Västra Götaland 41345 Sweden

**Keywords:** Suicide, Heart disease, Cardiac disease, Primary care, Somatic specialist care

## Abstract

**Background:**

Heart diseases are among the leading causes of disease burden worldwide with often a large impact on the lives of the affected individuals. The association between heart disease and depression is well-known and elevated suicide risk has been demonstrated in several types of heart disease. The aim of this study was to investigate what health care contacts individuals with heart disease who died by suicide had in the two years prior to death, compared to those without, and whether signs of mental health problems were recognised and addressed.

**Methods:**

As a part of a larger project, medical records from all care settings (primary care, hospital records etc.) for the last two years for all individuals who died by suicide in Sweden in 2015 were evaluated, *n* = 1,179. In this sub-study, we investigated healthcare contacts for the individuals with heart disease, *n* = 124, and compared these to those without, *n* = 1,055. We analysed the clinical characteristics of their last visit in all health care settings, and demographic and clinical factors contributing to recognition of depression when outside of psychiatric specialist care.

**Results:**

The individuals with heart disease had more healthcare contacts than those without during the last two years before death. During their final week of life, 58% were in contact with health care, compared to 32% for those without (*p* < .001). The contacts took place primarily in primary care and somatic specialist care. At the last doctor’s visit, 51% had symptoms of depression and/ or anxiety recorded, but less than 25% had an action taken due to this if the visit was in primary care or somatic specialist care. Suicide risk was assessed as elevated in 15%, but only a small proportion of these in primary care (3%) and somatic specialist care (4%).

**Conclusion:**

The individuals with heart disease had many healthcare contacts before dying by suicide, especially in primary care and somatic specialist care, yet their mental health problems were not sufficiently identified or treated. This points to a need to improve the recognition and treatment of depression in these settings to support suicide prevention in individuals with heart disease.

**Supplementary Information:**

The online version contains supplementary material available at 10.1186/s12875-026-03184-x.

## Background

Heart disease has a prevalence of approximately 5.5–11% globally [1, 2] and is often associated with chronic symptoms, reduced function and diminished quality of life [3–5]. Individuals with heart disease are two to three times more likely to develop major depression [6], which affects up to one-fourth of this population [7]. Treating depression in individuals with heart disease is considered important for improving both mental and cardiac health [7].

Several pathways have been proposed to explain the link between depression and heart disease. Both physiological mechanisms, such as hyperactivity of the hypothalamic-pituitary-adrenal axis (HPA), and behavioural mechanisms, such as negative lifestyle changes in the depressed individual, have been shown to have adverse cardiovascular effects [7]. Furthermore, psychological factors such as a perceived sense of loss of health or functional capacity associated with heart disease may contribute to depression in affected individuals [6]. Some symptom overlaps between conditions like congestive heart failure (CHF) and depression—such as loss of energy and impaired concentration—may also contribute to the underrecognition of either disorder in patients with both conditions [8].

An association between heart disease and suicide has been reported in several large registry-based studies [9–11], although it has been debated whether this is the case for heart disease in general [12]. The potential connection between heart disease and suicide has been shown most notably for myocardial infarction (MI) [13], acute coronary syndrome (ACS) [14] and CHF [10, 15]. In studies that have demonstrated an association, heart disease has been identified either as an independent risk factor [13, 14] or as a condition linked to suicide risk primarily through concurrent depression or other confounding factors [10, 16]. Whether caused by depression or not, an elevated prevalence of suicidality in individuals with heart disease has been reported, for example, in a large US primary care-based screening study that found that suicidal thoughts were significantly more common among patients with heart disease compared to those with no such history [14]. Similarly, a German multicentre study reported that 14% of cardiology patients (both in- and outpatients) had experienced suicidal thoughts in the past two weeks [17], with depression and severe anxiety identified as the primary contributing factors.

Although screening for depression and suicidal thoughts for individuals with heart disease has been recommended in clinical settings by the American Heart Association (AHA) [18], numerous barriers have been identified [19]. The extent to which individuals with heart disease who die by suicide have received treatment for mental illness remains unclear.

The aim of this study was to investigate the clinical characteristics of individuals with heart disease who died by suicide in Sweden with a particular focus on the identification and treatment of mental health conditions prior to death. The study sought to answer the following research questions: When and where did these individuals have contact with the healthcare system? Did this differ from those without heart disease? To what extent were mental health conditions, including suicidality, documented during these contacts? What factors were associated with the identification of depressive symptoms or a diagnosis of depression in this population? And finally, in cases where psychiatric problems were identified, what interventions or treatments, if any, were provided?

## Methods

### Design

This study is part of the nationwide project “Retrospective investigation of health care utilization of individuals who died by suicide in Sweden in 2015”. The study explored medical records from the two years preceding suicide, including records from primary care, somatic and psychiatric specialist care. The research team had access to medical records from all major healthcare providers in both the public and private sectors, as well as from smaller healthcare providers.

The research group developed a review template based on the Swedish Psychiatric Association’s guidelines for the assessment and treatment of suicidal patients [20]. The broader methodology of the nationwide study is described in detail by Bergqvist et al. [21]. In the present study, clinical data were drawn from individuals’ healthcare contacts across primary healthcare, specialist psychiatric and somatic healthcare, including variables such as symptoms, diagnoses, assessments and planned treatments, as outlined below.

### Data collection

The Swedish National Board of Health and Welfare provided data from the National Cause of Death Register, including personal identification numbers, dates of death and regions of residence for all registered residents who died by suicide between January 1 and December 31, 2015. Suicide was defined using ICD-10 codes X60–X84, intentional self-harm [22].

To review the medical records, the research team trained mental health professionals in each of Sweden’s 21 regions. The reviewers received a thorough one-day training session, covering potential difficulties, and had access to a manual as well as support from the researchers’ training team throughout the period of data extraction for any queries. They assessed the records of individuals residing in their respective regions. All data were collected in de-identified format and entered manually into structured templates tailored to each care setting: psychiatric specialist care, primary healthcare, and somatic specialist care.

### Study population/ setting

The total population of Sweden on December 31, 2015, was approximately 9.85 million [23]. The Swedish Cause of Death Register listed 1,179 confirmed suicides in Sweden that year. An additional seven cases were later added to the register after the initial data release and were therefore not included. One further case was excluded due to a pre-existing confidentiality agreement. The present study focuses on a defined subgroup of this population: individuals with a clinical diagnosis of heart disease. For the purpose of this study, heart disease was defined using ICD-10 codes I20–I25, I30–I39, I40–I49, and I50–I52 (see Supplement 1). A total of 124 individuals met these criteria (*n* = 124).

### Selected variables

The selected variables of interest in this study were:

- Age.
- Gender (male or female; for individuals who had transitioned, the chosen gender was used).
- Occupation (one or more of being employed, studying or participating in an employment agency project, as opposed to not being active in any of these categories. The latter could be the case if the individual was for example on sick leave or was unemployed without participating in an employment agency project etc.)
- Retired (Age-related).
- Marital status (married, cohabiting or neither).
- Previous suicide attempt.
- Healthcare contacts (visits or telephone contacts with any healthcare professional, including physicians, nurses, psychologists, counsellors or physiotherapists).
- Depressiveness or anxiety as noted by a physician at the last consultation in any care setting.
- Somatic symptoms and signs: cardiovascular symptoms, excluding hypertension and physical pain as noted by a physician at the last consultation.
- Elevated suicide risk as noted by a physician at the last consultation in any setting.
- Diagnoses (ICD-10 codes; multiple diagnoses allowed).
- Investigations, referrals, planned follow-ups and treatments at the last physician consultation with a GP or somatic specialist.
- Prescribed psychotropic medication (N05A, N05B, N05C, N06A, N06A, N06B, N06C) according to the Anatomical Therapeutic Chemical (ATC) classification, as listed in the medical record at the time of death.

Occupation, age-related retirement and marital status were based on the most recent notation in any case record.

### Statistical analyses

Categorical variables -such as background characteristics, psychiatric and somatic symptoms, history of suicide attempts, documented suicide risk, diagnoses, and planned treatments- were analysed using frequency distributions. Group comparisons for these variables were performed using chi-squared (χ²) tests.

Age, treated as a continuous variable, was compared between groups using independent samples *t*-tests and verified with a Mann-Whitney U test. To explore whether differences in age or gender could explain the elevated number of healthcare contacts among individuals with heart disease, we performed logistic regression analyses with adjustments for these variables.

In a second set of analyses, logistic regression was used to examine associations between selected independent variables—namely age, gender, a notation of anxiety, cardiovascular symptoms (excluding hypertension), physical pain, having a recent healthcare contact or a previous suicide attempt—and the dependent variable, defined as a binary outcome indicating the presence (yes/no) of either documented depressive symptoms or a clinical diagnosis of a mood disorder (ICD-10 codes F30–F39) during a visit to a physician in primary care or somatic specialist care in the last 6 months. This time frame was chosen to capture possible signs of a mood disorder preceding the individual’s decision to end their life by suicide. Individuals with neither depressive symptoms nor a mood disorder diagnosis served as the reference group. The logistic regression analyses were exploratory and performed as univariate models.

All statistical tests were two-sided, and results were considered statistically significant at a p-value < 0.05. Analyses were performed using SPSS Statistics version 28 [24].

## Results

### Characteristics of the study population

One tenth of the individuals who died by suicide in Sweden in 2015 had been diagnosed with heart disease (124 out of 1,179). Characteristics of the total study cohort and in subgroups with and without heart disease are shown in Table 1. The individuals with heart disease were considerably older than those without, and the proportion of males was higher in the heart disease group. Slightly more than one-third of all individuals were noted as being married or cohabiting, with no significant difference between those with and without heart disease. Less than half of the total group (44%) had an occupation, with a higher number of retired individuals in the older group with heart disease. Twenty-nine per cent of the total population had a recorded previous suicide attempt, with no significant difference between those with and without heart disease.

**Table 1:** Demographic characteristics and previous suicide attempts

	Total *n* = 1179 (*n*, %)	Heart Disease		*P*	df*
		Yes *n* = 124 (*n*, %)	No *n*= 1055 (*n*, %)		
Age (yrs, SD)	51.5 (± 19.1)	69.7 (± 15.2)	49.3 (± 18.4)	< 0.001	
Gender					
male	848 (71.9)	102 (82.3)	746 (70.7)	0.007	
female	331 (28.1)	22 (17.7)	309 (29.3)		1
Married/cohabiting**					
Yes	311 (36.4)	41 (42.7)	270 (35.6)	0.174	
No	543 (63.6)	55 (57.3)	488 (64.4)		1
Occupation***					
Yes	367 (44.3)	18 (18.4)	349 (47.7)	< 0.001	
No	179 (21.6)	19 (19.4)	160 (21.9)		
Retired (Age)	283 (34.1)	61 (62.2)	222 (30.4)		2
Previous suicide attempt ****	338 (28.7)	35 (28.2)	303 (28.7)	0.908	1

Differences between groups with and without heart disease were tested using independent samples t-tests (age), verified with the Mann-Whitney test *p*<.001, and chi-square analysis (all other variables)

*degrees of freedom

**n=854, missing 325 (27.6%); no data in medical records

***n=829, missing 350 (29.7%) no data in medical records

****as noted in any medical record during the last two years

### Healthcare contacts during the last two years of life

Ninety per cent of all 1,179 individuals who died by suicide in Sweden in 2015 had contact with healthcare services during the last two years of their lives. Proportions with health care contact were larger in the group with heart disease compared to the group without. This was observed across various time frames (Table 2). Tables 2, 3, 4 and 5 shows healthcare contacts during specified time periods prior to suicide, in patients with and without heart disease by care setting. These contacts could be with any health care staff member, e.g. physicians, nurses, psychologists, social counsellors, physiotherapists, occupational therapists etc., and in person or over the phone.

**Table 2:** Healthcare contacts during specified time periods prior to suicide. Any healthcare contacts during the last two years of life

	Total *n* = 1179 (*n*, %)	Heart Disease		*P*
		Yes *n* = 124 (*n*, %)	No *n*=1055 (*n*, %)	
24 months	1059 (89.8)	124 (100.0)	935 (88.6)	< 0.001
12 months	1010 (85.7)	123 (99.2)	887 (84.1)	< 0.001
3 months	867 (73.5)	114 (91.9)	753 (71.4)	< 0.001
4 weeks	699 (59.3)	97 (78.2)	602 (57.1)	< 0.001
1 week	428 (36.3)	72 (58.1)	356 (33.7)	< 0.001

**Table 3:** Contacts with primary care during the last two years of life

	Total *n* = 1179 (*n*, %)	Heart Disease		*P*
		Yes *n* = 124 (*n*, %)	No *n*=1055 (*n*, %)	
24 months	892 (75.7)	116 (93.5)	776 (73.6)	< 0.001
12 months	806 (68.4)	113 (91.1)	693 (65.7)	< 0.001
3 months	505 (46.6)	88 (71.0)	462 (43.8)	< 0.001
4 weeks	346 (29.3)	61 (49.2)	285 (27.0)	< 0.001
1 week	156 (13.2)	34 (27.4)	122 (11.6)	< 0.001

**Table 4:** Contacts with somatic specialist care during the last two years of life

	Total *n* = 1179 (*n*, %)	Heart Disease		
		Yes *n* = 124 (*n*, %)	No *n*=1055 (*n*, %)	
24 months	733 (62.2)	115 (97.2)	618 (58.6)	< 0.001
12 months	639 (54.2)	110 (88.7)	529 (50.1)	< 0.001
3 months	405 (34.4)	81 (65.3)	324 (30.7)	< 0.001
4 weeks	235 (21.5)	59 (47.6)	194 (18.4)	< 0.001
1 week	116 (9.8)	37 (29.8)	79 (7.5)	< 0.001

**Table 5:** Contacts with psychiatric specialist care during the last two years of life

	Total *n* = 1179 (*n*, %)	Heart Disease		*P*
		Yes *n* = 124 (*n*, %)	No *n*=1055 (*n*, %)	
24 months	614 (52.1)	56 (45.2)	558 (52.9)	0.103
12 months	570 (48.3)	53 (42.7)	517 (49.0)	0.187
3 months	493 (41.8)	44 (35.5)	449 (42.6)	0.131
4 weeks	384 (32.6)	30 (24.2)	654 (33.6)	0.035
1 week	242 (20.5)	22 (17.7)	220 (20.9)	0.417

Individuals with heart disease had a higher proportion of healthcare contacts in primary care compared to those without heart disease (Table 3). Among all individuals who died by suicide in Sweden in 2015, 76% had visited primary care at least once during the last two years. The heart disease group consistently showed higher rates of primary care contact across all examined time periods.

62% of the total cohort had contact with somatic specialist care during the same period (Table 4), with the heart disease group again demonstrating higher contact rates at every time point.

Higher age and female gender were both significantly associated with healthcare utilization. However, even after adjusting for these demographic factors in regression analyses, individuals with heart disease continued to show significantly greater odds of having healthcare contact. This was primarily driven by elevated contact rates in both primary care and somatic specialist care, suggesting that the increased service use in this group cannot be fully explained by age or gender alone (see Supplementary Material II for details).

However, when examining the rates of psychiatric specialist care contacts (Table 5), there were no statistically significant differences between individuals with and without heart disease during the last two years, with one exception. In the last four weeks, a lower proportion of the individuals with heart disease had contact with psychiatric services compared to those without.

**Fig. 1:**
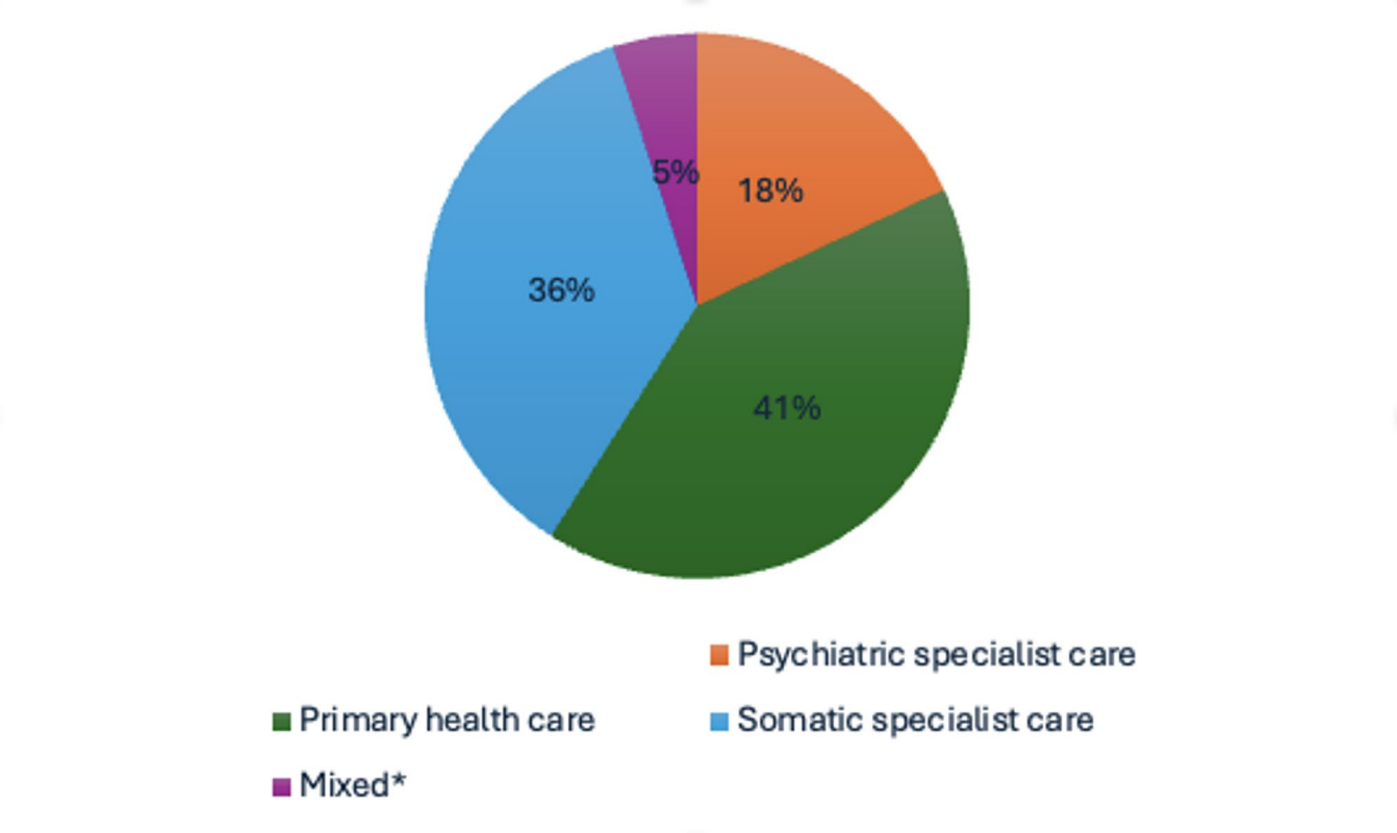
Setting for last healthcare contact in persons with heart disease who died by suicide. *Mixed: Last contact in two different healthcare settings on the same day. Mixed primary care/somatic specialist care 2%, mixed primary care/psychiatric specialist care 1%, mixed psychiatric specialist care/somatic specialist care 2%

Among individuals with heart disease, the majority had their final contact in either primary care or somatic specialist services, a contact that could, as in Tables 2, 3, 4 and 5 above, be with any health care staff member, and in person or over the phone. Only about one-fifth of these individuals had their last healthcare contact within psychiatric specialist care, while the remainder had their final contact in primary healthcare or somatic specialist care. This is illustrated in Fig. 1.

For individuals with heart disease, the average time between their last healthcare contact of any kind and death was 24 days (median 5 days, range 0 to 493 days). The average time between primary care contact and death was 67 days (median 27 days, range 0 to 650 days). For somatic specialist care, the average was 81 days (median 27 days, range 0 to 724 days), and for psychiatric specialist care 66 days (median 21 days, range 0 to 565 days).

For individuals with heart disease, the average time since their last visit to a physician was 116 days before death (median 106 days, range 0 to 585 days). Specifically, the average time since their last visit to a primary care physician was 120 days (median 60 days, range 0 to 650 days), to a physician in somatic specialist care 117 days (median 31 days, range 0 to 724 days), and to a physician in psychiatric specialist care 105 days (median 42 days, range 1 to 676 days).

### Identification of mental health issues at last physician consultation (heart disease group only)

As shown in Table 6 40% of the individuals with heart disease were noted to have symptoms of depression at their last physician consultation in one or more of the care settings; anxiety was noted in a third. Just over half (51%) of the 124 individuals had a recorded notation of depressive and/or anxiety symptoms in any healthcare setting, including 28% in primary care and 25% in somatic specialist care. A diagnosis of F30-F39 (mood disorders) was noted for 32% of the individuals at their last visit with a physician, and 22% had an F40-F48 diagnosis (neurotic, stress-related, somatoform disorders). Further, a physician noted an elevated suicide risk for 14% of these individuals, which was similar to the 15% observed in individuals without heart disease during their last physician contact.

**Table 6:** Identification of mental health problems in individuals with heart disease at last physician consultation before suicide, by care setting. (*n*, %)

	In at least one specialty including primary care *n* = 124 *n* (%)	Primary care *n* = 116 *n* (%)	Somatic specialist care *n* = 115 *n* (%)	Psychiatric specialist care *n* = 56 *n* (%)
Depressive symptoms	50 (40.3)	24 (20.7)	21 (18.3)	29 (51.8)
Anxiety symptoms	40 (32.3)	21 (18.1)	17 (14.8)	18 (32.1)
Mood disorder diagnosis*	39 (31.5)	20 (17.2)	15 (13.0)	26 (46.4)
Neurotic etc. disorder diagnosis**	27 (21.8)	16 (13.8)	6 (5.2)	9 (16.1)
Elevated suicide risk	17 (13.7)	3 (2.6)	5 (4.3)	11 (19.6)

* F30–F39 Mood disorders

** F40–F48 Neurotic, stress-related, somatoform disorders

### Actions taken due to mental health concerns at the last visit to a physician (heart disease group only)

**Fig. 2:**
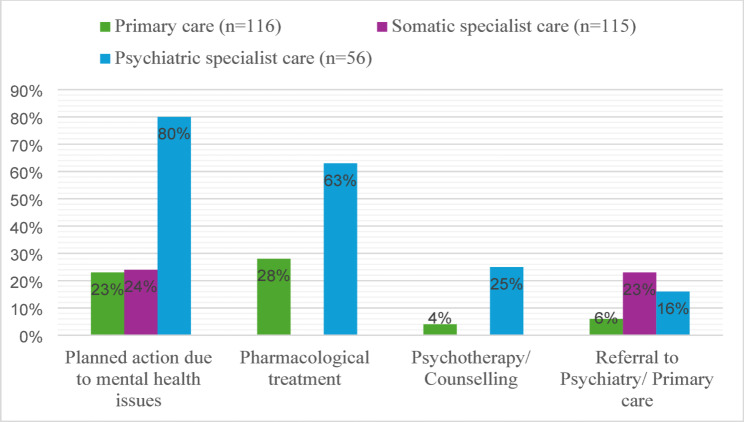
Treatment and planned actions due to mental health issues at the last physician consultation* in each healthcare setting prior to suicide in individuals with heart disease, *n* = 124. * An individual could have been seen by a physician in one or more healthcare settings. ** No information was available on pharmacological treatment or psychotherapy/counselling from somatic specialist care

As illustrated in Fig. 2 80% of individuals with heart disease had a notation indicating that an action (as a referral, prescription, planned psychotherapy or other measure, new or continuation of a previous one) was taken in response to psychiatric concerns during their last visit to a physician in psychiatric specialist care. In contrast, this figure was 23% and 24%, respectively, for the last visit to a physician in primary healthcare and somatic specialist care. Additionally, 6% of individuals in primary care were referred to psychiatric specialist services, and 23% of the individuals in somatic specialist care were referred to either psychiatric services or primary care for their psychiatric symptoms.

Pharmacological treatment for psychiatric issues was prescribed for 63% of the individuals in psychiatric specialist care, compared to 28% in primary healthcare. Furthermore, counselling or psychotherapy was planned or ongoing for 25% of individuals in psychiatric specialist care and for 4% in primary healthcare.

### Factors associated with the identification of depressive symptoms or mood disorders

Table 7 presents data on individuals with heart disease who had their last visit to a physician in either primary healthcare or somatic specialist care within six months prior to death (*n* = 119). Of these, 46 individuals (37%) were identified as having either depressive symptoms or a clinical diagnosis of a mood disorder (ICD-codes F30-F39) during their visit.

**Table 7:** Results from univariate logistic regression analyses, with the binary outcome depressive symptoms/Mood disorder diagnosis (Yes/No) prior to suicide in individuals with cardiac disease who were in contact with a physician in primary care or somatic specialist care during the last six months of life (*n* = 199), crude odds ratio (OR) with 95% confidence interval (95% CI)

	All *n* = 119	Depressive symptoms/ Mood disorder diagnosis		Univariate OR (95% CI)	*p*
		No (*n* = 73)	Yes (*n* = 46)		
Age at death, yrs, SD	70.0 ± 14.5	70.5 ± 15.8	69.1 ± 13.6	0.99 (0.97–1.02)	0.633
Gender, n (%)					
Male	97 (81.5)	59 (80.8)	38 (82.6)	0.89 (0.34–2.32)	0.807
Female	22 (18.5)	14 (19.2)	8 (17.4)		
Physical pain	57 (47.9)	34 (46.6)	23 (50.0)	1.15 (0.55–2.40)	0.714
Anxiety symptoms	31 (26.1)	13 (17.8)	18 (39.1)	2.97 (1.28–6.89)	0.011
Substance use diagnosis	12 (10.1)	5 (6.8)	7 (15.2)	2.44 (0.73–8.21)	0.149
Cardiovascular symptoms*	69 (58.0)	42 (57.5)	27 (58.7)	1.049 (0.496–2.217)	0.901
4 weeks or less between the last visit to physician** and death	74 (62.2)	43 (58.9)	31 (67.4)	1.442 (0.666–3.123)	0.353
Previous suicide attempt	33 (27.7)	17 (23.3)	16 (34.8)	1.757 (0.778–3.965)	0.175

*hypertension excluded

** in primary care or somatic specialist care

The analysis explored potential associations between the identification of depressive symptoms or a mood disorder diagnosis and various clinical and demographic factors. Only the presence of anxiety symptoms showed a significant association, with an odds ratio (OR) of 2.97 (95% CI, 1.28–6.89, *p* = .011).

Other factors, including age, gender, physical pain, substance abuse, cardiovascular symptoms, timing of healthcare contact in relation to death, and previous suicide attempts, did not demonstrate a significant association with the outcome (the identification of depressive symptoms or a mood disorder diagnosis).

## Discussion

### Main findings

Among suicide decedents in Sweden, those with heart disease were more likely to have physician contact during the year that preceded death compared to those without this conditon. More than half (58%) of those in the heart disease group had a healthcare contact during their final week of life. Most care contacts occurred in primary care or somatic specialist care, although one-third had contact with psychiatric specialist care during the last three months.

Symptoms of anxiety and/or depression were documented in 51% of cases at the last physician visit in the individuals with heart disease (28% in primary healthcare and 25% in somatic healthcare). However, notations of elevated suicide risk were rare in the medical records. Fewer than one in four individuals with heart disease had any action taken regarding mental health issues at their final visit to a physician in primary care or somatic specialist care.

### Healthcare contacts

The proportions of healthcare contacts prior to suicide observed in our study were relatively high; 99% of individuals with heart disease were in contact with healthcare at some point during the last year, and 78% had such contacts within the last four weeks. We found no studies for direct comparison, but note that our figures are somewhat higher than those reported for any healthcare contacts in a longitudinal US study (83% during the last year, and 50% within the last four weeks) [25]. A French study found that 61% visited a physician in the last month, and 34% in the last week [26]. As morbidity increases with age, healthcare contacts are more frequent in older populations [27]. Given that the mean age of the individuals with heart disease in our study was nearly 20 years higher than those without, the very high levels of healthcare contacts observed in our study were not unexpected. However, the findings remained constant after adjusting for age. The relatively high levels of healthcare contacts may also be partly explained by our inclusion of not only visits to a physician but also contacts with other healthcare staff members as well as telephone consultations.

Almost half of the individuals with heart disease had contact with primary healthcare during the final four weeks, and a similar proportion were in contact with somatic specialist care. In contrast, only about one in four had contact with psychiatric specialist care during the same period. The majority had their last contact with primary healthcare, only approximately one in five individuals had their last healthcare contact with psychiatric specialist care. This aligns with previous research showing primary healthcare and somatic specialities as the most common points of contact in the final months before death by suicide, as demonstrated by Ahmedani et al. [25] and an older large review by Luoma et al. [28]. A more recent study from Wales also found that the last healthcare contacts before death by suicide were most commonly with primary healthcare [29].

### Identification of mental health problems at the last physician consultation

Depressive symptoms were noted in the medical records of 40% of individuals with heart disease at their last physician consultation in any care context, while anxiety was recorded in 32%. Notably, half of these individuals had a documentation of either or both symptoms. Despite these relatively frequent notations of depressive or anxious symptoms slightly fewer than one-third of all those with heart disease received a mood disorder diagnosis. While it can be assumed that some persons had subthreshold depression, which is associated with increased risk of both fatal and non-fatal suicidal behavior in older adults [30, 31], our finding does suggest a potential gap between symptom recognition and formal psychiatric diagnosis in clinical practice. Comparable findings emerge in international studies. For instance, a Slovenian study also indicated a similar pattern of low recognition of psychiatric disorders at the final primary care visits before suicide, with psychiatric diagnoses recorded in 30% of the individuals. Though the frequency of mood diagnoses was significantly higher compared to 3% in living controls [32], it nevertheless underscores possible under-diagnosis in primary care settings. A similar trend is observed in a study from Taiwanese context, where psychiatric diagnoses at the last medical visit were rare, occurring in only about one in five individuals at their last visit to a non-psychiatric physician within their final month before suicide [24]. The authors of that study noted that pronounced somatic symptoms might have contributed to physicians’ greater focus on physical rather than mental health problems, a dynamic potentially relevant to our findings as well.

Explicit notations of elevated suicide risk in our study were rare, primarily found in psychiatric specialist care, and infrequently in primary care or somatic specialist care settings. This aligns with findings from a UK study, where only 15% were assessed as having elevated suicide risk at their final general practitioner visit before suicide [33]. Several factors could explain these low rates of recorded elevated suicide risk: patients might not have been actively suicidal at their medical consultations, might have experienced suicidal thoughts but were hesitant to disclose them, or healthcare providers may have inadequately assessed or understood the patient’s suicide risk. Reflecting this challenge, recent discourse in the field has increasingly highlighted the limitations of predictive risk assessments and advocates shifting towards a more therapeutic and proactive clinical approach. This approach emphasises the importance of identifying and addressing modifiable risk factors rather than attempting to predict future suicidal behaviour [34].

### Identification of depressive symptoms or a mood disorder in individuals with cardiac disease

Known risk factors such as higher age, male gender, physical pain, substance abuse or a previous suicide attempt did not associate with being identified as having depressive symptoms or a mood disorder at the last visit to a physician in primary care or somatic specialist care during the final six months of life. Nor was there an association with cardiovascular symptoms or with the visit occurring shortly before death. The only factor that showed an association was anxiety. Individuals with anxiety were three times more likely to also be identified with depressive symptoms or a mood disorder. This is not surprising given the frequent comorbidity between anxiety and depression. In our recent report from this Swedish nationwide suicide study, anxiety symptoms were noted in the case records of half of all individuals who had a physician consultation during the last week of life [35]. That the other risk factors did not show an association with the identification of depression might also not be surprising given their high frequency in the population, with the exemption of a known previous suicide attempt where one in four of the individuals with heart disease is a higher frequency than the general population [36]. It can be suspected that the physicians in primary care and somatic specialist care, where the individuals had their most health care contacts, might not always have been aware of the information of a previous suicide attempt even if it was recorded somewhere in the individuals’ medical records. It is also possible that they were aware of previous behavior but assumed that the individual was already adequately treated in psychiatric specialist care. This could reflect the so-called siloing of healthcare which can be detrimental to patients [37].

### Actions and treatments in response to mental health issues at the last visit

More than one in five individuals with heart disease who had a final consultation in primary care or somatic specialist care had an action taken due to mental health issues. In psychiatric care, this proportion was four out of five. Psychopharmacological treatment (including antidepressants) was the most common intervention, recorded in 63% of cases in psychiatric specialist care and 28% in primary care. Counselling or psychotherapy was rarely provided -only 4% of individuals with heart disease received this in primary care, though it was somewhat more common in psychiatric specialist care (25%). A referral to psychiatric specialist care was made for one in five individuals when combining visits in primary care and somatic specialist care, though more might have been in need of a referral.

Though not directly comparable, the proportion receiving pharmacological treatment in primary care aligns with another Swedish study of individuals aged 75 and over who died by suicide, which found that one-third had filled an antidepressant prescription during the last three months of life [38]. This is a higher proportion than in the general population, where 11% had an antidepressant prescription filled during the last year, according to the Swedish National Board of Health and Welfare, with the highest rate—over one in five—among those aged 75 and over [39].

### Clinical implications

Many suicide decedents with heart disease saw a physician shortly before death. These care contacts took place primarily in primary care and somatic specialist settings. Such contacts offer important opportunities for suicide prevention, yet mental health symptoms are often insufficiently addressed. However, results should not be interpreted as support for universal screening or routine referral of all cardiac patients with psychological symptoms. Instead, they emphasize the need for targeted, context-sensitive assessment of suicidality, especially among patients with repeated contacts, persistent psychological symptoms, or signs of distress in non-psychiatric care. Different levels of assessment may be warranted depending on clinical setting and urgency, and some patients may already be in psychiatric treatment. The fact that approximately half of the individuals had been noted to have depressive symptoms and/or anxiety at their last physician visit and one third were in contact with psychiatric specialist services during the last three months, yet still died by suicide, also underscores the potential need for a more active approach to identify and manage suicidality. One such approach is the therapeutic suicide assessment and risk management advocated in a recent article by Hawton et al. [34].

Training primary care physicians and nurses in the recognition and treatment of depression [40], the use of brief interventions [41], and access to collaborative care models [42, 43] may strengthen suicide prevention efforts in this group. Furthermore, as studies indicate that antidepressant treatment plays a significant role in preventing suicide [44, 45], perhaps more individuals in with heart disease could have benefited from such treatment at the primary care level. While most of these interventions would be recommendable for the general population, the individuals with heart disease, who have a known higher risk of depression and suicidality, might benefit from them even more.

In summary, clinicians in primary and somatic care should not be expected to conduct comprehensive psychiatric assessments, but rather to recognise warning signs, initiate appropriate conversations, and ensure timely referral and follow-up when necessary. Developing practical, role-specific clinical guidance may provide valuable support for such decision-making in everyday practice.

## Strengths and limitations

The relatively small sample size of the subgroup with diagnosed heart disease is a limitation of this study in comparison with large registry-based studies and could impact the generalisability of the findings. On the other hand, a strength of the study is the detailed information regarding healthcare utilisation before death by suicide, made possible by the medical record review design. This gives access to detailed and context-bound information. Access to medical records was also very good [21]. The sample of individuals with heart disease in the study is an older population group with a physical disorder, and the study’s findings might be generalisable to other similar groups.

The symptom variables were recorded as binary yes/no responses based on whether symptoms were noted during the last physician visit. This means that an individual might have had symptoms that were not noticed or that were noticed but not recorded. The variable of depressive symptoms/mood disorder diagnosis was gathered from the medical records with no further assessment by the researchers. Likewise, the variable of elevated suicide risk was based on a binary yes/no question, as recorded by the physician in the medical record at the final consultation, at any care setting. No further information was available on how this conclusion was reached. Due to the nature of our data, we cannot determine how many individuals were actually assessed for suicidal ideation or suicide risk.

No inter-rater reliability (IRR) testing was conducted for medico-legal reasons, which is a limitation, but training and support from the research group were provided to the examiners to ensure consistency in data extraction. Data were entered manually, and input errors may occur. To mitigate this problem, members of the research team reviewed the entered data for the first cases, to identify potential issues. This troubleshooting led to some modifications in the structured templates and record reviewers were notified.

The subgroup analysis (*n* = 124; regression sample *n* = 119) limited power for low-frequency variables such as substance use diagnoses, resulting in wide confidence intervals. Thus, estimates have limited precision and must be interpreted cautiously. Non-significant associations should be considered inconclusive rather than evidence of no effect.

A further limitation is that the data stem from healthcare contacts from 2013 to 2015. Clinical practice, documentation routines, and awareness of suicidal tendencies may have changed since then. However, the study focuses on structural patterns of healthcare contacts and recognition of mental health problems, which have proven to be relatively stable over time. The results should be interpreted as informative about underlying mechanisms rather than current prevalence estimates.

## Conclusion

Although individuals with heart disease who died by suicide had many healthcare contacts, especially in primary care and somatic specialist care, their mental health problems and suicidality were not sufficiently recognised or treated. There is a clear need to improve the identification and treatment of depression and anxiety in individuals with heart disease within primary care and somatic health care to strengthen suicide prevention efforts.

## Supplementary Information

Supplementary Material 1.

Supplementary Material 2.

## Data Availability

Relevant data are available from the corresponding author upon reasonable request.
