# Supplementary material for: A retrospective cohort study of clinical characteristics and healthcare contacts in Sweden prior to suicide in individuals with heart disease
Source: BMC Prim Care. 2026 Feb 9;27:66. doi: 10.1186/s12875-026-03184-x (PMC12918212; doi:10.1186/s12875-026-03184-x)
Supplement: Supplementary file 1 — Supplementary Material 1. [file 12875_2026_3184_MOESM1_ESM.docx]

Supplement 1:

ICD codes

I20-I25 Ischemic heart diseases

I20 Angina pectoris

I21 Acute myocardial infarction

I22 Subsequent myocardial infarction

I23 Certain current complications following acute myocardial infarction

I24 Other acute ischemic heart diseases

I25 Chronic ischemic heart disease

I30-I52 Other forms of heart disease

I30 Acute pericarditis

I31 Other diseases of pericardium

I32 Pericarditis in disorders classified elsewhere

I33 Acute and subacute endocarditis

I34 Nonrheumatic mitral valve disorders

I35 Nonrheumatic aortic valve disorders

I36 Nonrheumatic tricuspid valve disorders

I37 Nonrheumatic pulmonary valve disorders

I38 Endocarditis, valve unspecified

I39 Endocarditis and heart valve disorders in diseases classified elsewhere

I40 Acute myocarditis

I41 Myocarditis in diseases classified elsewhere

I42 Cardiomyopathy

I43 Cardiomyopathy in diseases classified elsewhere

I44 Atrioventricular and left bundle-branch block

I45 Other conduction disorders

I46 Cardiac arrest

I47 Paroxysmal tachycardia

I48 Atrial fibrillation and flutter

I49 Other cardiac arrhythmias

I50 Heart failure

I51 Complications and ill-defined descriptions of heart disease

I52 Other heart disorders in diseases classified elsewhere
