# Supplementary material for: A retrospective cohort study of clinical characteristics and healthcare contacts in Sweden prior to suicide in individuals with heart disease
Source: BMC Prim Care. 2026 Feb 9;27:66. doi: 10.1186/s12875-026-03184-x (PMC12918212; doi:10.1186/s12875-026-03184-x)
Supplement: Supplementary file 2 — Supplementary Material 2. [file 12875_2026_3184_MOESM2_ESM.docx]

Supplement II

*Odds ratios for having recorded health care contacts during the last year prior to suicide in individuals with/without heart disease, and the association of this for age and gender, crude (Univariate) and adjusted (multivariate) odds ratio (OR) with 95% confidence interval.*

|  | Total sample  n=1179  n (%) | Diagnosis of Heart Disease:  Yes No  n=124 n=1055  n (%) n (%) | | Crude OR  (95% CI) | *p-value* | Adjusted OR  (95% CI) | *p-value* |
| --- | --- | --- | --- | --- | --- | --- | --- |
| Health care contacts during the last week | 428 (36.3) | 72 (58.1) | 356 (33.7) | 2.72 (1.86-3.97) | <0.001 | 3.28 (2.16-4.98) | <0.001 |
| Age |  |  |  |  |  | 1.08 (1.06-1.09) | <0.001 |
| Gender |  |  |  |  |  | 2.11 (1.25-3.55) | .005 |
| Health care contacts during the last 4 weeks | 699 (59.3) | 97 (78.2) | 602 (57.1) | 2.70 (1.74-4.41) | <0.001 | 2.92 (1.82-4.68) | <0.001 |
| Age |  |  |  |  |  | 1.07 (1.06-1.09) | <0.001 |
| Gender |  |  |  |  |  | 2.04 (1.22-3.41) | .007 |
| Health care contacts during the last 3 months | 867 (73.5) | 114(91.9) | 753 (74.1) | 4.57 (2.36-8.85) | <0.001 | 4.44 (2.23-8.82) | <0.001 |
| Age |  |  |  |  |  | 1.07 (1.06-1.08) | <0.001 |
| Gender |  |  |  |  |  | 2.08 (1.24-3.47) | .005 |
| Health care contacts during the last 12 months | 1010 (85.7) | 123(99.2) | 887 (54.1) | 23.30 (3.23-167.86) | .002 | 20.89 (2.85-152.97) | .003 |
| Age |  |  |  |  |  | 1.07 (1.05-1.08) | <0.001 |
| Gender |  |  |  |  |  | 2.20 (1.31-3.68) | .003 |

*Odds ratios for having recorded primary care contacts during the last year prior to suicide in individuals with/without heart disease, and the association of this for age and gender, crude (univariate) and adjusted (multivariate) odds ratio (OR) with 95% confidence interval.*

|  | Total sample  n=1179  n (%) | Diagnosis of Heart Disease:  Yes No  n=124 n=1055  n (%) n (%) | | Crude OR  (95% CI) | *p-value* | Adjusted OR  (95% CI) | *p-value* |
| --- | --- | --- | --- | --- | --- | --- | --- |
| Primary care contacts during the last week | 156 (13.2) | 34 (27.4) | 122 (11.6) | 2.89 (1.87-4.47) | <0.001 | 2.06 (1.27-3.33) | .003 |
| Age |  |  |  |  |  | 1.07 (1.5-1.08) | <0.001 |
| Gender |  |  |  |  |  | 2.02 (1.21-3.37) | .007 |
| Primary care contacts during the last 4 weeks | 346 (29.3) | 61 (49.2) | 285 (27.0) | 2.62 (1.79-3.81) | <0.001 | 1.71 (1.14-2.58) | .010 |
| Age |  |  |  |  |  | 1.07 (1.05-1.08) | <0.001 |
| Gender |  |  |  |  |  | 1.93 (1.16-3.22) | .011 |
| Primary care contacts during the last 3 months | 505 (46.6) | 88 (71.0) | 462 (43.8) | 3.14 (2.09-4.41) | <0.001 | 2.10 (1.36-3.24) | <0.001 |
| Age |  |  |  |  |  | 1.07 (1.05-1.08) | <0.001 |
| Gender |  |  |  |  |  | 2.05 (1.23-3.43) | .006 |
| Primary care contacts during the last 12 months | 806 (68.4) | 116(93.5) | 776 (73.6) | 5.37 (2.85-10.10) | <.001 | 3.72 (1.93-7.17) | <.001 |
| Age |  |  |  |  |  | 1.07 (1.05-1.08) | <0.001 |
| Gender |  |  |  |  |  | 2.12 (1.27-3.57) | .005 |

*Odds ratios for having recorded somatic specialist care contacts during the last year prior to suicide in individuals with/without heart disease, and the association of this for age and gender, crude (univariate) and adjusted (multivariate) odds ratio (OR) with 95% confidence interval.*

|  | Total sample  n=1179  n (%) | Diagnosis of Heart Disease:  Yes No  n=124 n=1055  n (%) n (%) | | Crude OR  (95% CI) | *p-value* | Adjusted OR  (95% CI) | *p-value* |
| --- | --- | --- | --- | --- | --- | --- | --- |
| Somatic care contacts during the last week | 116 (9.8) | 37 (29.8) | 79 (7.5) | 5.25(3.36-8.22) | <0.001 | 5.87 (3.36-9.29) | <.001 |
| Age |  |  |  |  |  | 1.07 (1.06-1.09) | <0.001 |
| Gender |  |  |  |  |  | 2.12 (1.25-3.62) | .006 |
| Somatic care contacts during the last 4 weeks | 235 (21.5) | 59 (47.6) | 194 (18.4) | 4.03 (2.74-5.92) | <0.001 | 3.51 (2.30-5.35) | <.001 |
| Age |  |  |  |  |  | 1.07 (1.05-1.08) | <0.001 |
| Gender |  |  |  |  |  | 2.06 (1.22-3.47) | .007 |
| Somatic care contacts during the last 3 months | 405 (34.4) | 81 (65.3) | 324 (30.7) | 4.25 (2.87-6.29) | <0.001 | 3.07 (2.02-4.67) | <0.001 |
| Age |  |  |  |  |  | 1.06 (1.05-1.08) | <0.001 |
| Gender |  |  |  |  |  | 2.01 (1.20-3.38) | .008 |
| Somatic care contacts during the last 12 months | 639 (54.2) | 110(88.7) | 529 (50.1) | 7.81 (4.42-13.80) | <.001 | 6.03 (3.34-10.87) | <.001 |
| Age |  |  |  |  |  | 1.06 (1.05-1.08) | <0.001 |
| Gender |  |  |  |  |  | 2.14 (1.28-3.61) | .004 |
